# A novel procedure for stabilization of azide in biological samples and method for its determination (HS-GC-FID/FID)

**DOI:** 10.1038/s41598-021-95104-5

**Published:** 2021-07-30

**Authors:** Olga Wachełko, Marcin Zawadzki, Paweł Szpot

**Affiliations:** 1 Institute of Toxicology Research, 45 Kasztanowa Street, 55093 Borowa, Poland; 2 Department of Forensic Medicine, Wroclaw Medical University, 4 J. Mikulicza-Radeckiego Street, 50345 Wroclaw, Poland

**Keywords:** Medical research, Analytical chemistry

## Abstract

Sodium azide is an old poison with toxicity comparable to potassium cyanide. It would seem to be completely forgotten however, between 2000 and 2020, the number of intentional ingestions and murders committed with sodium azide significantly increased. Furthermore, due to its extreme instability, sodium azide is difficult to detect, which poses an additional risk when used to commit a crime. In this study, the epidemiology of sodium azide exposures between 1920 and 2020 was investigated. For the determination the azide concentration in biological samples, a simple, precise and selective headspace gas chromatography method (HS-GC-FID/FID) was developed and fully validated. The limit of quantification was 0.65 µg/mL; and the limit of detection was 0.35 µg/mL; precision and accuracy did not exceed 20%. The stability study was conducted for various biological fluids (urine, bile, blood, gastric content) for 91 days in the refrigerator (4 °C) and the method for stabilization of azide was presented. The addition of a mixture of borax and sodium fluoride (*w/w* 3:1) to the test tubes can stabilize this poison. The described unique technique of collecting the biological samples poses a great potential for azide detection in clinical and toxicology laboratories even long time after human exposure to this substance.

## Introduction

Sodium azide (NaN_3_) is a white crystalline powder, which transforms into volatile hydrazoic acid (NH_3_) in an acidic medium. Due to its antibacterial properties, sodium azide is widely used as a preservative in aqueous laboratory reagents. It has been also investigated as a herbicide, insecticide and fungicide. The most common signs and symptoms in acute sodium azide intoxications are headache, dizziness, sweating and hypothermia with metabolic acidosis. A massive release of nitric oxide after ingestion leads to hypotension and arrhythmia^1^. The earliest methods for sodium azide determination are spectrophotometric^2,3^ and volumetric^4^, which appeared in the early 1960s. Over time, a number of chromatographic methods have been developed: high performance liquid chromatography with ultra-visible detector (HPLC-UV)^5^, high performance liquid chromatography with diode array detector (HPLC-DAD)^6–8^, ion chromatography (IC)^9^, headspace gas chromatography with nitrogen phosphorus detector (HS-GC-NPD)^10^, gas chromatography with nitrogen phosphorus detector (GC-NPD)^11^, and gas chromatography coupled with mass spectrometry (GC–MS)^12–14^.

Sodium azide has been determined in several biological fluids (blood, bile, urine, gastric content) and tissues (brain, lung, liver, kidney, heart, muscle)^6–8,11^. However, there is an analytical problem described by Kruszyna et al.^9^, Ohmori et al.^13^ and Le Blanc-Louvry et al.^8^, related to high azide instability in biological samples and its rapid degradation. This phenomenon can significantly underestimate intoxication statistics. Kinetic studies of azide degradation indicate that at the temperature of 0 °C the half-life (*t*_1/2_) is 12 days, while at room temperature (20 °C) half-life (*t*_1/2_) is only 4.5 days^9^. Due to the extreme lability of this anion, in the fatal suicide case described by Le Blanc-Louvry et al.^8^ azide was not found in the blood sample. Intoxication was finally confirmed on the basis of analysis of other biological fluids. For this reason, toxicological examination of postmortem samples may be misleading because, in fact, a negative result does not always indicate the lack of sodium azide poisoning. To date, the exact mechanism of azide degradation in biological samples is unknown. It is supposed that rapid decrease in concentration is related to the oxidative effect of hemoglobin (O_2_-Hb)^13^ or to the enzyme activity and acidification of biological fluids, which leads to the conversion of azide anion into hydrazoic acid^8^.

### Epidemiology of intoxications

**Figure 1:**
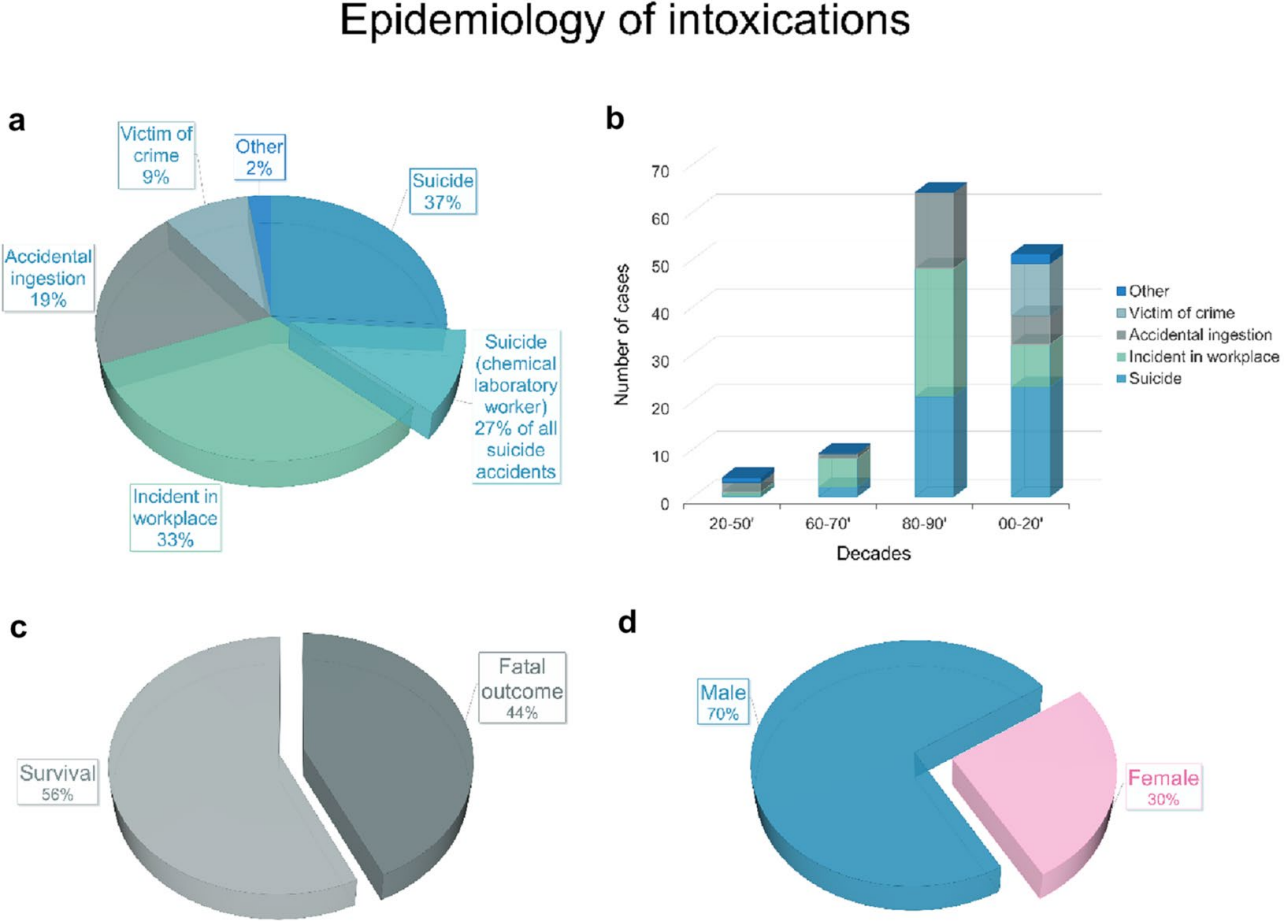
Epidemiology of sodium azide intoxications (**a**) circumstances of exposures; (**b**) exposition between 1920 and 2020 divided into decades; (**c**) outcome of poisoning; (**d**) the relationship between the number of men and women committing suicide by sodium azide ingestion.

Of the cases reported so far, the epidemiology of intoxications was developed on the basis of records obtained by entering keywords “sodium azide intoxication” and “sodium azide poisoning” in PubMed and Google Scholar websites. In the epidemiology investigation, 62 articles were found describing 130 cases of exposure to sodium azide over the last century. The cases were divided according to the circumstances (Fig. 1a), the decade (Fig. 1b), the outcome of the incident (Fig. 1c) and the sex of the individuals who attempted suicide with the use of sodium azide (Fig. 1d). Of all the cases reported so far, as many as 37% concerned the use of sodium azide in suicide attempts. One-third of these issues were related to chemical laboratory workers who had easy access to this substance. Workplace incidents associated with unintentional exposure to sodium azide represented 33% of the total. These included mouth pipetting and, thus, accidental consumption of a sodium azide solution^15^ as well as inhalation of this substance during improper unloading of pallets at the airport^16^. Moreover, there were also cases of accidental poisoning, which included intoxication with sodium azide after drinking a beverage from a contaminated pot in the restaurant^17^ and at the university^18^. A similar situation took place in the storeroom adjacent to the laboratory where one chemical technician filled a kettle of distilled water that had been previously treated with sodium azide to prevent bacterial growth^19^. Another reported case of accidental exposure to sodium azide resulting in hypotension of nine patients occurred in a dialysis center^20^. In turn, 9% of all reported poisonings were related with intentional administration of sodium azide with murderous intentions^21^. The last group of cases (2%) included exposures after the use of sodium azide during self-experimentation. The majority of these incidents occurred in the 1920s–1950s. However, this harmful laboratory practice has survived until now and even in 2014, French et al.^22^ reported a case of a young technician who consumed 100 mg of sodium azide salt to investigate what the maximum dose of poison is needed to induce vomiting.

The chart presented in Fig. 1b shows that from the early ’80s until now there has been a significant increase in exposures to sodium azide in comparison with previous decades. Particularly alarming is the persistently high number of intentional ingestions of this substance for suicidal purposes. Moreover, since 2000, a huge growth in the number of murders committed with sodium azide is also visible. The fatal outcome involved almost half of all the described sodium azide intoxications (Fig. 1c). However, taking into account only suicides, death was the result in 92% of cases, while the majority (70%) of all suicides with the use of sodium azide were committed by men (Fig. 1d). Epidemiology of intoxications indicates that the problem of unintentional or intentional use of sodium azide is still a current issue. As Bruin et al. stated in their recent paper, in the last few years, the attention for sodium azide as a suicide drug in the Netherlands is rising, and even in 2020 two fatal cases of intentional ingestion were reported^11^. Furthermore, easy access to this poison on websites such as Amazon^®^^23^ poses a real danger these days. The reported ranges of sodium azide concentration in authentic caseworks were as follows: blood, 2.6–262 µg/mL; bile, 21.4–1283 µg/mL; intestinal contents, 429–7360 µg/g; gastric contents, 8.8–58,900 µg/g; and liver, 6.1–14 µg/g. In kidney, brain and lungs, sodium azide was found in concentration of 205 µg/g, 2.7 µg/g and 17.6 µg/g, respectively^6^.

This paper aims to apply a novel headspace gas chromatographic method with dual column/dual flame ionization detector (HS-GC-FID/FID) for the determination of azide in biological material. A new procedure of collecting the samples in order to stabilize azide concentration in biological fluids for forensic purposes has also been described. Determination of azide was based on the derivatization of the azide ion [N=N=N^–^] with propionic anhydride [(CH_3_CH_2_CO)_2_O], leading to the formation of ethyl isocyanate [CH_3_CH_2_–N=C=O].

## Methods

### Chemicals

Water (ChemSolve, LC–MS) was purchased from Witko (Łódź, Poland); propionitrile (≥ 99% purity, internal standard, ISTD), propionic anhydride (≥ 99% purity, transforming reagent), ethyl isocyanate (98% purity, reaction product), sodium tetraborate (borax; 99% purity), sodium fluoride (≥ 99% purity) were purchased from Sigma-Aldrich (Steinheim, Germany); Sodium azide salt was purchased from Merck (Darmstadt, Germany); The aqueous internal standard solution of propionitrile was prepared in concentration of 100 µg/mL. The standard aqueous solution used to create the calibration curve was prepared by dissolution of 10 mg of sodium azide salt (6.5 mg of azide ions) in 1 mL LC–MS grade water. The working standard solutions were prepared by dilution of stock solution with water. Depending on the combined cation (e.g. Na^+^, K^+^, NH_4_^+^) with azide anion, the total weight of the salt may be different, therefore all dilutions have been prepared based on the concentration of azide ions, which were further derivatized and quantified. Due to high toxicity of sodium azide and propionitrile, all operations were carried out in a fume cupboard. Aqueous standard solutions of ethanol, methanol, acetone and isopropanol were purchased from Cerilliant (Round Rock, Texas, USA), *n*-propanol (99.8% purity) from CPAchem (Stara Zagora, Bulgaria) and all were used in the selectivity study. The stock solution and standard solutions were stored at − 20 °C, while ISTD was stored at 4 °C.

### Biological material

Drug-free blank blood samples used for the development and validation of the method as well as for stability studies were obtained from Regional Blood Donation Center. Urine, bile and gastric content samples (used in stability study and in validation process) as well as blood samples (used in selectivity study) were sent to our laboratory for toxicological analysis. The samples were screened prior to spiking to ensure that they were free from sodium azide.

### Ethical statement

All procedures performed in this study were in accordance with the 1964 Declaration of Helsinki and its later amendments or comparable ethical standards. This article does not contain any studies with living human participants or animals performed by any of the authors. Blood, urine, bile and gastric content collections from decedents were made by judicial authorities during the prosecution, and the samples were sent to our institute for toxicological analysis at their request. In accordance with the law in force, permission was obtained from the prosecutor's office to utilize these biological samples for further toxicological examinations and to publish the obtained results. The study was approved by the Ethical Committee of the Medical University of Wroclaw, Poland (No. 333/14).

### Working solutions, calibration curve, and quality control samples

For standard calibration curve (in fivefold), 0.2 mL of blood was added to a headspace vial (10 mL, Alwsci Technologies Shaoxing, China) and mixed with sodium azide aqueous solution to create ten concentration levels of azide ions (0.65, 1, 3, 6.5, 10, 15, 20, 30, 45 and 65 µg/mL). Next, 0.1 mL of the internal standard aqueous propionitrile solution (100 µg/mL) and 10 µL of propionic anhydride were added. The vials were immediately sealed with headspace caps (aluminum cap: butyl rubber/PTFE, Polygen, Gliwice, Poland) and vortex mixed for 5 s. Quality control samples were prepared by spiking blank human whole blood to yield final concentration of 0.65 (low QC), 10 (medium QC) and 45 (high QC) µg/mL.

### Sample procedure

As in the calibration procedure, 0.2 mL of biological fluid was added to a headspace vial. Next, 0.1 mL of the internal standard aqueous propionitrile solution (100 µg/mL) and 10 µL of propionic anhydride were added. The vials were immediately sealed with headspace caps and vortex mixed for 5 s.

### Apparatus

A Shimadzu GC-2010 Plus AF IVD (Kyoto, Japan) equipped with an advanced flow controller (AFC), a split/splitless injector (SPL) and two Flame Ionization Detectors (FID) was used in this study. An static headspace sampler (HS-20 Shimadzu, Kyoto, Japan) was used for sample preparation and introduction into the GC through a single SPL. Effluent from the HS-20 was divided between two columns: Zebron-BAC1, 0.32 mm × 30 m × 1.8 µm, (Phenomenex, Torrance, California, USA) and Zebron-BAC2, 0.32 mm × 30 m × 1.2 µm, (Phenomenex, Torrance, California, USA) using a SilFlow^®^ micro-fluidic platform (SHI-980–10,593, Trajan, Ringwood, Victoria, Australia) at a 1:1 ratio. Each column was connected to a separate FID and analyzed simultaneously. Operating headspace gas chromatography with dual column and dual flame ionization detector parameters are presented in Table 1.

**Table 1:** Dual column HS-GC-FID/FID operating parameters.

HS-20	GC-2010 Plus
Oven temperature: 75 °C	Carrier gas: He
Sample line temperature: 200 °C	First column: Zebron ZB-BAC1 0.32 mm × 30 m × 1.80 µm
Transfer line temperature: 200 °C	Second column: Zebron ZB-BAC2 0.32 mm × 30 m × 1.20 µm
Shaking level: 1	Column temperature: 40 °C/hold time: 6 min
Multi injection count: 1	Up to 150 °C/rate: 25.0/hold time: 3 min
Pressurize gas pressure: 70.0 kPa	Column flow: 2.57 mL/min
Equilibrating time: 5 min	Linear velocity: 40 cm/s
Pressurizing time: 0.5 min	Total flow: 55.1 mL/min
Pressure equlib. time: 0.1 min	FID1 and FID 2 temperature: 240 °C
Load time: 0.5 min	FID1 and FID 2 makeup flow: 30 mL/min
Load equilibrating time: 0.1 min	FID1 and FID 2 H2 flow: 40 mL/min
Injection time: 1 min	FID1 and FID 2 air flow: 400 mL/min
Needle flush time: 1 min	APC1 pressure: 70 kPa
Injection mode: split	Data acquisition stop time: 6 min
Sampling time: 1 min	Total program time: 13.40 min

### Validation

Validation was carried out according to SWGTOX recommendations^24^ for all biological fluids (whole blood, urine, bile and gastric content) tested in the stability study. Evaluated parameters of the method included examination of linearity, precision and accuracy, carryover, limit of detection and quantification, recovery, matrix effect and selectivity.

#### Linearity

Linearity was evaluated by analysis of sodium azide working solutions with human blood in final azide ions concentrations of 0.65, 1, 3, 6.5, 10, 15, 20, 30, 45 and 65 µg/mL. Linear calibration model was applied. The coefficient of determination (*R*^2^) was determined. According to the acceptance criteria used, the coefficient of determination should meet the condition: *R*^2^ ≥ 0.995.

#### Precision and accuracy

The intra-day and inter-day precision and accuracy were estimated by replicating analysis (n = 5) of QC samples at three concentration levels: 0.65, 10, and 45 µg/mL. The results from both detectors (FID1 and FID2) were averaged. To determine precision and accuracy values for the method, standard deviation (SD), relative standard deviation (RSD), relative error (RE) and coefficient of variation (CV) were calculated.

#### Carryover

To investigate the carryover, three samples without analytes were analysed after a calibration sample at the azide concentration of 65 µg/mL (highest calibration level). Unacceptable carry over was when peak area ratio in a zero sample after analysis of a sample containing a high concentration of azide ion exceeded 20% of the area ratio observed for the LOQ samples.

#### The LOQ and the LOD

The limit of quantification (LOQ) was defined as the concentration at which the relative standard deviation (RSD%) and relative error (RE%) does not exceed 20% and 15%, respectively^25^. The limit of detection (LOD) was considered to be the lowest concentration of the sample for which the signal to noise ratio met the condition at least: S/N ≥ 3.

#### Recovery and matrix effect

The recovery (n = 5) was evaluated at each of the three different concentrations 0.65, 10, and 45 µg/mL. The recovery (in percent) was determined by comparing concentration of analytes in spiked blank biological sample versus concentration of standard solutions. Matrix effect (in percent) was calculated using equation described by Chambers et al.^26^.

### Selectivity

#### Endogenous substances

Fifteen different types of blood samples were divided into three groups depending on the toxicological case type: low ethyl alcohol content (below 0.5‰), high ethyl alcohol content (more than 4‰) and with confirmed ketoacidosis. The effect of endogenous substances from a matrix was investigated in order to determine if there are any interference peaks which could affect the analysis results. For this purpose, each sample was prepared in two repetitions: the first by adding 0.2 mL of blood and 10 µL of propionic anhydride, the second: the same procedure but with the addition of 0.1 mL of internal standard.

#### Other volatile substances

Selectivity was studied for other volatile compounds routinely determined in toxicological analysis (methanol, ethanol, acetone, isopropanol and *n*-propanol).

### Stability study

In order to examine whether neutralization of the biological matrix will stabilize sodium azide, we decided to use borax, which has strongly alkaline properties and is routinely used in azide analysis as a mobile phase in liquid chromatography^9^ or as the environment of derivatization reaction in GC techniques^13^. The optimal amount of borax for the whole biological sample volume, was tested for five blank blood samples (5 mL) spiked with sodium azide solution to the final azide ions concentration of 50 µg/mL, which were collected in tubes containing 5, 10, 15, 20 and 30 mg/mL of borax, respectively. The samples were analyzed immediately after preparation as well as one month later. The results showed that even the smallest amount of borax is able to stabilize azide, but due to different initial pH values of the biological fluids, the addition of 15 mg of borax per 1 mL of sample was considered the most appropriate.

Different types of urine, bile, blood and gastric content samples were firstly analyzed to exclude the presence of azide. Then, each type of biological matrix was mixed and spiked with a standard aqueous solution of sodium azide to the final azide ions concentration of 50 µg/mL. The pooled biological fluids were collected in 4 types of tubes: (1) without any preservative agent, (2) with sodium fluoride (5.0 mg per 1 mL of biological fluid), (3) with borax (15.0 mg per 1 mL of biological fluid) and (4) with a mix of borax and sodium fluoride (*w/w* 3:1; 15.0 mg of borax and 5.0 mg of sodium fluoride per 1 mL of biological fluid). The samples were left in the refrigerator (4 °C) for 3 months. Analysis of the samples was performed immediately after the addition of azide ions and again on days: 1, 3, 5, 7, 11, 15, 31, 41, 61 and 91. The temperature of the refrigerator (4 °C) was chosen because it is a standard storage condition for biological material in forensic toxicology and clinical laboratories.

## Results

### Validation process

**Figure 2:**
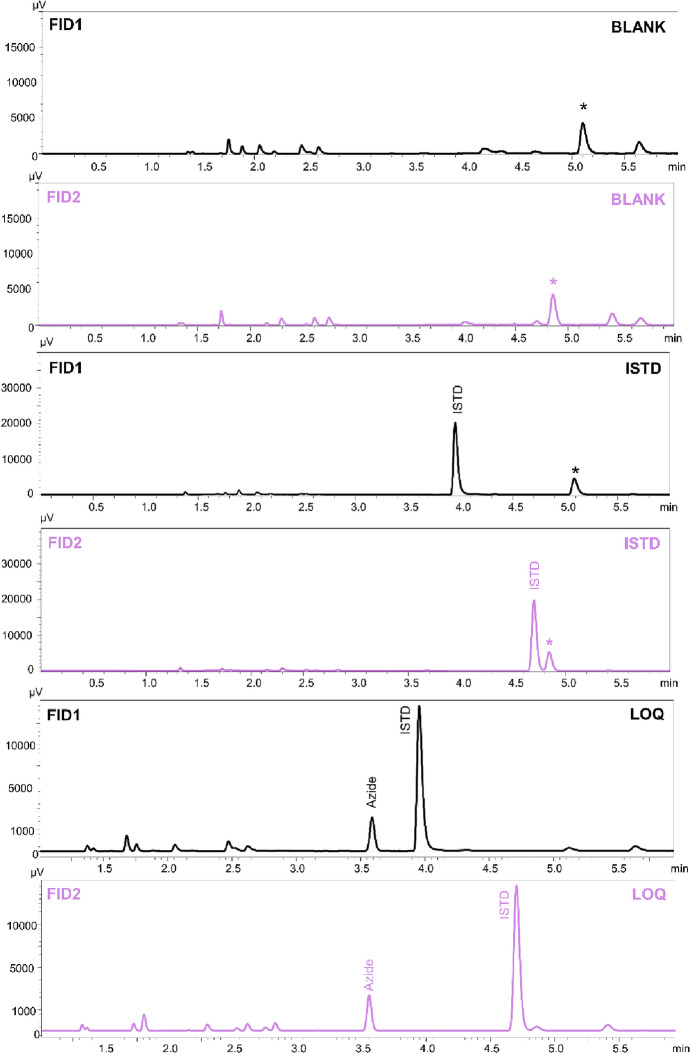
Chromatograms of blank blood sample, ISTD, and azide at concentration of LOQ with ISTD. The unreacted derivatization reagent (propionic anhydride) has been marked with an asterisk. The black line illustrates the results from FID1 and the violet line from FID2.

**Figure 3:**
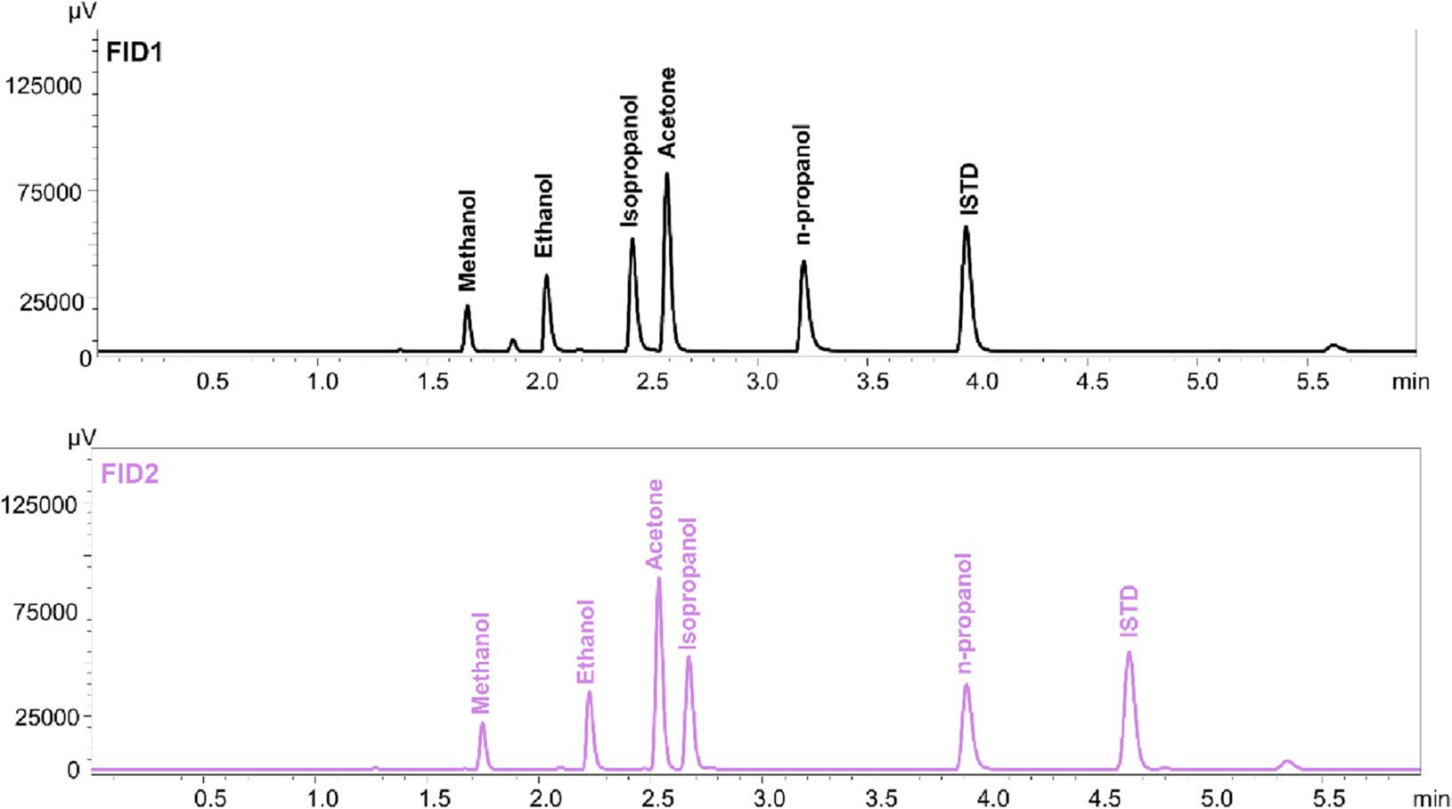
Chromatograms of other volatile compounds routinely determined in forensic toxicology (methanol, ethanol, acetone, isopropanol, *n*-propanol) with ISTD (propionitrile). Black line is illustrating result from FID1 and violet line from FID2.

**Figure 4:**
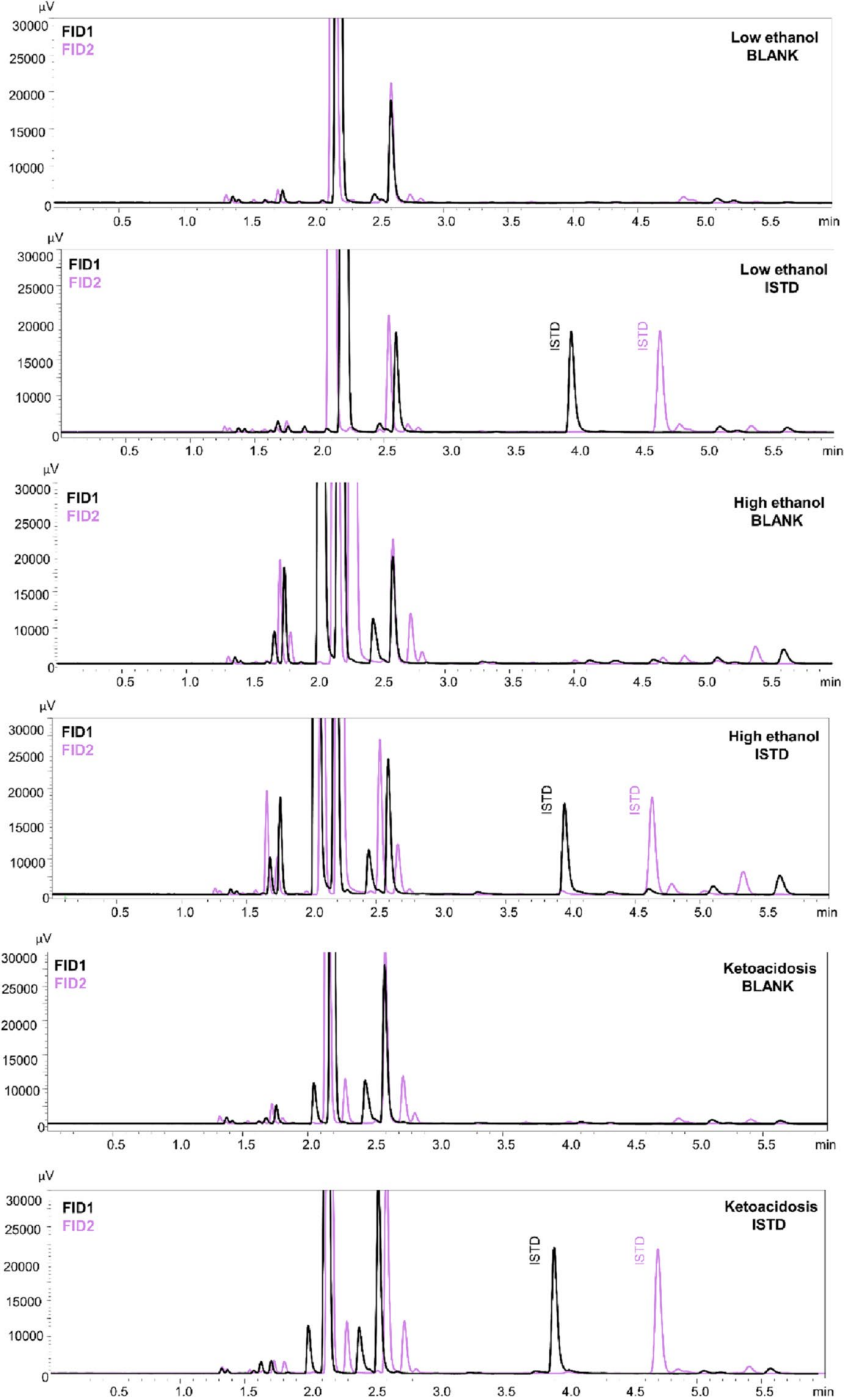
Selectivity of the method with regard to endogenous substances contained in the samples in case of low ethyl alcohol content (below 0.5‰), high ethyl alcohol content (more than 4‰) and ketoacidosis. Black line is illustrating result from FID1 and violet line from FID2.

In the described method, very good validation parameters were achieved. The value of the coefficient was 0.999 (*R*^2^). The intra- and inter-day validation results are presented in Table 2. The limit of quantification (LOQ) was 0.65 µg/mL. Chromatograms of the blank sample, ISTD and azide at a concentration of LOQ with ISTD are presented in Fig. 2. The retention time of propionitrile (ISTD) on the first column (Zebron-BAC1) was 3.95 min., on the second (Zebron-BAC2) 4.70 min., while retention time of ethyl isocyanate (azide derivative) was 3.59 min. and 3.55 min., respectively. Chromatograms of the blank blood sample and ISTD show unreacted propionic anhydride, which retention time on the first column was 5.10 min. and 4.87 min. on the second. In the presence of azide ions, propionic anhydride becomes a derivatization reagent in the ethyl isocyanate formation. The analysis of the sample spiked with a lower concentration (0.35 µg/mL) showed that the signal from analyte was also visible (S/N = 5) but the RSD exceeded 20%. Because lower concentration samples were not analyzed, 0.35 µg/mL was determined as the limit of detection (LOD) of the method. Furthermore, there were no substances carried over between samples. Recovery and matrix effect values in blood samples were in the range 87.8–89.0% and 11.0–12.2%, respectively. In other biological fluids recoveries and matrix effects were in the range 73.3–88.4% and 11.6–26.7%, respectively. Selectivity studies have shown that the method is suitable for the analysis of biological samples containing other volatile compounds (methanol, ethanol, isopropanol, acetone, *n*-propanol) (Fig. 3). Moreover, no substances from the matrix in different types of cases affected the determination of the azide ion. All peaks were well separated and no endogenous substances interfered with the retention time of analyte or internal standard. Chromatograms are presented in Fig. 4.

**Table 2:** Validation results for various biological matrix.

Biological matrix	Concentration of azide [µg/mL]	Intra-day (n = 5)				Inter-day (n = 5)				Recovery [%] (n = 5)	Matrix effect [%] (n = 5)
		Precision			Accuracy	Precision			Accuracy		
		SD	RSD%	CV%	RE%	SD	RSD%	CV%	RE%		
Whole blood	0.65	0.1	10.0	9.0	− 9.6	0.1	9.6	9.3	− 3.9	87.8	12.2
	10	1.4	9.9	9.3	− 6.9	1.1	7.0	7.0	0.1	89.0	11.0
	45	6.2	8.8	8.8	0.3	7.2	10.0	10.0	2.6	88.5	11.5
Urine	0.65	0.2	17.2	18.5	7.2	0.2	14.8	15.5	4.4	88.4	11.6
	10	1.5	10.7	7.0	− 7.4	1.5	10.7	7.0	− 7.4	84.3	15.7
	45	4.6	6.7	6.6	− 0.5	5.5	8.0	7.9	− 2.1	80.8	19.2
Bile	0.65	0.1	11.6	12.3	5.8	0.1	11.4	11.7	2.7	88.4	11.6
	10	1.5	10.7	10.8	− 7.4	1.5	10.7	11.3	− 7.4	83.9	16.1
	45	6.4	8.8	9.2	4.5	7.0	9.6	10.0	3.7	83.4	16.6
Gastric content	0.65	0.2	17.6	19.7	12.0	0.2	16.6	17.8	7.6	78.4	21.6
	10	1.5	10.7	3.4	− 7.4	1.5	10.7	4.1	− 7.4	75.5	24.5
	45	6.7	9.2	9.6	4.3	7.0	10.1	10.0	− 0.2	73.3	26.7

*SD* standard deviation, *RSD%* relative standard deviation, *CV%* coefficient of variation, *RE%* relative error.

### Stability of azide in biological fluids

**Figure 5:**
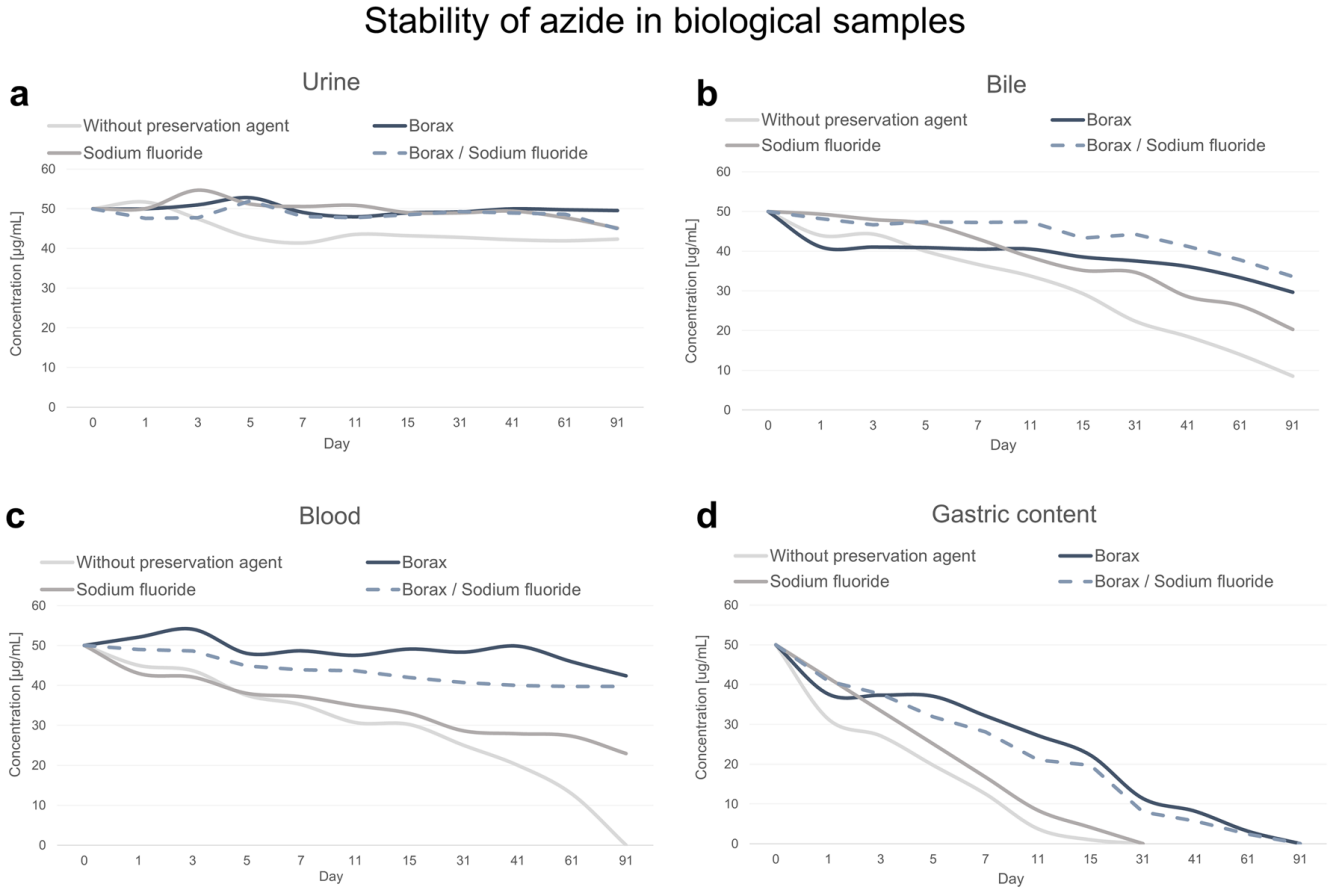
Stability of azide in biological samples—(**a**) urine; (**b**) bile; (**c**) blood; (**d**) gastric content—collected in four types of tubes: without any preservation agent, with borax, with sodium fluoride, and with mix of borax and sodium fluoride (*w/w* 3:1).

The results of stability studies indicate the highest decrease of azide in bile, blood and gastric content collected in tubes without any preservative agent and in tubes with sodium fluoride. In the case of urine, azide demonstrated relative stability regardless of the type of tube into which the material was collected. Moreover, the addition of borax as the only preservative and in the mixture with sodium fluoride stabilized the azide ions in biological fluids. In the case of urine, bile and blood, the percentage of azide residue in comparison with the initial content was: 99%, 59% and 85% for samples taken for borax alone and 90%, 67% and 80% for tubes with a mix of borax and sodium fluoride (*w*/*w* 3:1). In the gastric content, due to the alkalinization of the matrix, the degradation process was slightly delayed. However, the procedure of the borax addition facilitated detection of the azide content in the sample even after three months (Fig. 5).

## Discussion

A novel method described in this paper allows for simultaneous determination of azide and ethyl alcohol with its congeners (methanol, isopropanol, acetone and *n*-propanol) in biological samples. Sample pre-treatment is fast and simple, while the derivatization occurs in the autosampler (headspace oven) during the vial heating (75 °C by 5 min). Additionally, the use of two columns with different polarities significantly increased selectivity and helped to avoid overlapping peaks. This practice is essential for reliable analysis, especially in the case of methods in which the retention time is the only factor identifying a given substance^5–8,10,11^. The determination of azide based on the reaction with propionic anhydride was previously applied by Meatherall et al.^10^ for HS-GC-NPD analysis of biological samples, and by Wachełko et al.^27^ for HS-GC-FID/FID analysis of pharmaceutical products. However, pharmaceutical methods developed for the study of tablets are not suitable for the determination of the substances in biological material due to the more complex matrix of biological specimens (especially postmortem). Consequently, methods developed for clinical and toxicological investigations are required to exhibit much greater sensitivity and selectivity. They must also be re-validated to check the usefulness of the method for the intended purpose. The methods presented above had a similar azide derivatization procedure, however Meatherall et al.^10^ utilized different detector, and Wachełko et al.^27^ used a completely different samples and matrix to develop the method for azide determination. The headspace technique coupled with gas chromatography and flame ionization detection is one of the most widespread methods for routine determination of volatile compounds in both clinical and forensic toxicology laboratories. However, to our knowledge, the HS-GC-FID/FID method for azide detection in biological samples has not been reported to date.

Liquid and gas chromatographic methods are definitely, among the most reliable methods for the determination of azide content in biological samples. A comparison of these methods is presented in Table 3. Chromatographic, as opposed to spectrophotometric and volumetric methods, are characterized by high levels of sensitivity, selectivity and accuracy, which are essential for reliable verification of azide poisoning. However, these methods usually require the derivatization of azide with the use of benzoyl chloride^7,8^, and 3,5-dinitrobenzoyl chloride^5,6^ for liquid chromatography, and pentafluorobenzyl bromide^12–14^ for gas chromatography. All these procedures are complicated and time consuming (average time of derivatization is about 30 min.). In addition, the gas chromatography coupled with mass spectrometry methods usually involve the use of quaternary amine salts (phase transfer catalysts) to transport azide ions from the aquatic medium (biological fluid) into the organic phase. The next step is the addition of derivatization reagent (PFB-Br), which binds the ions and forms the determined product (PFB-N_3_). The most widespread phase transfer catalysts are tetrabutylammonium sulfate (TBAS) and tetradecyldimethylbenzylammonium chloride (TDMBA)^12,14^. However, according to the Kudo et al.^14^ report, the use of quaternary amines in the long term causes damage of chromatographic column. For successful elimination of the phase transfer catalyst before injection of the sample into GC, the organic supernatant must be purified with the use of the solid-phase extraction (SPE) step on the strong cation exchange (SCX) column. For this reason, the procedure becomes complicated and requires multi-stage preparation of the tested sample.

**Table 3:** Comparison of chromatographic methods for determination of azide in biological samples.

No.	Tested biological fluids (volume)	Matrix of calibration curve	Method	Derivatization reagent	Linear range [µg/mL]	LOD [µg/mL]	LOQ [µg/mL]	Precision	Accuracy	Recovery [%]	Year	References
1.	Plasma (5000 µL)	–	HPLC-UV	3,5-Dinitrobenzoyl chloride	–	0.01	–	–	–	97–103	1982	^5^
2.	Blood Bile Gastric content (1000 µL)	Water	HPLC-DAD	3,5-Dinitrobenzoyl chloride	0.5–5	0.08	–	4.0^a^	–	–	1995	^6^
3.	Blood Bile Gastric content (1000 µL)	Blood	HPLC-DAD	Benzoyl chloride	0.5–5	0.2	0.5	3.2–14.5^a^	1.2–19.2	103–109	1996	^7^
4.	Blood Bile Serum Urine Gastric content (1000 µL)	–	HPLC-DAD	Benzoyl chloride	–	0.1	–	–	–	–	2012	^8^
5.	Blood (1000 µL)	Blood	IC	–	0.1–10	0.03	–	1.8^b^	–	83–85	1997	^9^
6.	Blood urine (200 µL)	Blood/urine	GC–MS	Pentafluorobenzyl bromide	0.07–13	0.03	–	2.6–9.6^a^	–	50–90	2000	^12^
7.	Blood (200 mg) ^c^	Blood	GC–MS	Pentafluorobenzyl bromide	2–50	0.1	–	3.3–13.0^a^	–	–	2014	^13^
8.	Blood (200 µL)	Blood	GC–MS	Pentafluorobenzyl bromide	0.2–21	0.08	–	4.3–9.1^d^	− 4.8–0.6^e^	87–96	2018	^14^
9.	Plasma Blood (100 µL)	Plasma/blood	HS-GC-NPD	Propionic anhydride	0.04–20	0.01	0.04	5.6^a^	–	–	2009	^10^
10.	Plasma Serum Blood Urine (200 µL)	Plasma	GC-NPD	Propionic anhydride	1–100	0.09	1.0	0.7–20.4^a^	− 7.1–11.6^e^	–	2020	^11^

*LOD* limit of detection, *LOQ* limit of quantification, *HPLC-UV* high performance liquid chromatography with ultra-visible detection, *HPLC-DAD* high performance liquid chromatography with diode array detection, *IC* ion chromatography with conductivity detection, *GC–MS* gas chromatography coupled with mass spectrometry, *GC-NPD* gas chromatography with nitrogen phosphorus detection, *HS-GC-NPD* headspace gas chromatography with nitrogen phosphorus detection, *HS-GC-FID/FID* headspace gas chromatography with flame ionization detection (dual column/dual detector).

Parameters expressed as: ^a^coefficient of variation CV [%]; ^b^standard deviation SD [%]; ^c^weighed of the sample; ^d^relative standard deviation RSD [%]; ^e^relative error RE [%].

In previously reported gas chromatographic methods, very low limits of quantification (up to 0.04 µg/mL for HS-GC-NPD analysis) and wide range of linearity (up to 100 µg/mL for GC-NPD analysis) were achieved^10,11^. However, a HS-GC-FID/FID analysis is sufficient to determine azide in a wide range of lethal concentrations reported in the literature (0.65–65 µg/mL) and in different types of biological matrix (blood, bile, urine, gastric content). In case of higher azide concentrations, the biological sample should be appropriately diluted for analysis. Furthermore, the technique presented in this paper reduces the biological sample volume up to 200 µL and the quantification of azide is achieved with similar sensitivity to liquid chromatographic methods (which, in comparison, require the use of 1000–5000 µL of sample)^5–9^. The precision and accuracy values of our method are comparable to those previously developed. Moreover, a very good recovery was achieved (87–89%) for the full range of azide concentrations in blood (small, medium and high QC). In comparison, Kage et al.^12^, using the GC–MS method, achieved a maximum recovery of 50% for azide in the blood matrix.

In acute intoxications, average postmortem blood sodium azide concentrations are about 50 µg/mL. However, the reported sodium azide concentrations range from 2.6 to 262 µg/mL^28^. Such an enormous deviation in concentrations may partly be the result of the high instability of azide ions in the postmortem biological material and different periods of time between judicial examination of the corpse and toxicological analysis. The epidemiology of intoxications seems to prove that the introduction of chromatographic methods since the early 1980s, which are much more sensitive and selective than previous ones, has significantly increased the amount of reported azide poisonings. However, the real number of all intoxications may still be underestimated due to the high instability of this substance and the difficulties with its detection. Some previous research has suggested the use of low temperatures, − 20 °C, − 30 °C and − 70 °C, to stabilize azide concentration in biological samples. However, this solution is problematic for two reasons. First of all, storing biological specimens in special freezers is not a routine procedure in many toxicology laboratories around the world. Secondly, even after the application of such recommendations, it is not possible to fully stabilize the azide content before its degradation. According to the researchers statement, at temperatures of − 20 °C and − 30 °C, the azide is stable from 3 to 7 days^13,14^, while in − 70 °C up to 49 days^11^. Consequently, temperature is not the only factor determining the rapid decrease of this substance in biological fluids. The studies performed by Ohmori et al.^13^ on the stability of azide in plasma and red blood cells showed that sodium azide is stable in the first fraction as opposed to the second one. For this reason, it has been suggested that the azide instability in whole blood is caused by hemoglobin (O_2_-Hb), which rapidly oxidizes the azide anion. However, this may not be the only mechanism leading to the degradation of this substance. The stability study performed in this paper for other biological materials, such as bile or stomach content, indicates that sodium azide is not stable in any of the samples. Its concentration decreased regardless of the type of matrix. Furthermore, the faster the pH of the body fluid fell, the more rapid the degradation reaction was. In the blood samples over the time, hydrogen ions contained in the cells are released as a result of hemolysis, while the bile consists mainly of fatty acids and phospholipids, which significantly affect the pH. In turn, in the gastric content, which is the most acidic biological fluid, the azide was the most unstable. Sodium azide in urine did not decrease, similarly to the tested plasma samples in the stability study described by Ohmori et al. This phenomenon could be caused by the fact that both materials are not exposed to such rapid acidification as other samples. Furthermore, after the addition of sodium tetraborate (which has strong alkaline properties) to the samples, the azide was stabilized in most of the biological fluids. This proves that, indeed, one of the mechanisms leading to azide degradation is the progressive acidification of the sample. Therefore, neutralization of pH of the biological material is a technique which can stabilize the azide concentration (up to 85% of initial concentration in whole blood by 91 days in 4 °C). This fact is especially important in the case of postmortem blood samples, which are usually hemolyzed, making it potentially difficult to obtain the plasma.

## Conclusion

A novel headspace gas chromatography method (HS-GC-FID/FID) for azide determination in biological samples has been evaluated and fully validated. The method described in this paper is sensitive and precise, while the sample preparation procedure is fast and simple, and does not require complex analytical pre-treatment of biological material. Due to the high selectivity of the method, it can be successfully applied for routine analysis of azide concentration along with the other volatile compounds (such as methanol, ethanol, isopropanol, acetone and *n*-propanol), determined in both clinical and forensic toxicology departments.

### New procedure for collecting biological samples

The use of borax has stabilized azide in biological fluids. Samples taken for forensic toxicology purposes are frequently collected in tubes with sodium fluoride, which inhibits the process of glycolysis and slows down the formation of endogenous ethyl alcohol. For this reason, if sodium azide poisoning is suspected, the best method of collecting biological fluids might be the addition of a mixture of borax and sodium fluoride (*w/w* 3:1; 15.0 mg of borax and 5.0 mg of sodium fluoride per 1 mL of biological fluid) to the test tubes. Standard preservative agents in the commercial test tubes are sodium fluoride, EDTA, and heparin, however the described mix of borax with sodium fluoride poses a great potential for azide detection in clinical and toxicology laboratories even long time after human exposure to this substance.

## References

[1] Kurt TL, Klein-Schwartz W, Hall AH, Isom GE, Rockwood GA (2015). Azide poisonings. Toxicology of Cyanides and Cyanogens: Experimental, Applied and Clinical Aspects.

[2] Anton A, Dodd JG, Harvey AE (1960). Spectrophotometric determination of azide with ferric perchlorate. Anal. Chem..

[3] Tayyab S, Ali MK (1995). Alkaline azobilirubin color reaction to determine sodium azide. Clin. Chem..

[4] Koźlicka-Gajdzińska H, Brzyski J (1966). A case of fatal intoxication with sodium azide. Arch. Toxikol..

[5] Swarin SJ, Waldo RA (1982). Liquid chromatographic determination of azide as the 3,5-dinitrobenzoyl derivative. J. Liq. Chromatogr..

[6] Lambert WE, Piette M, van Peteghem C, de Leenheer AP (1995). Application of high-performance liquid chromatography to a fatality involving azide. J. Anal. Toxicol..

[7] Marquet P (1996). Analytical findings in a suicide involving sodium azide. J. Anal. Toxicol..

[8] Le Blanc-Louvry I (2012). Suicidal sodium azide intoxication: An analytical challenge based on a rare case. Forensic Sci. Int..

[9] Kruszyna R, Smith RP, Kruszyna H (1998). Determining sodium azide concentration in blood by ion chromatography. J. Forensic Sci..

[10] Maetherall R, Palatnick W (2009). Convenient headspace gas chromatographic determination of azide in blood and plasma. J. Anal. Toxicol..

[11] Bruin MAC (2021). Toxicological analysis of azide and cyanide for azide intoxications using gas chromatography. Basic Clin. Pharmacol. Toxicol..

[12] Kage S, Kudo K, Ikeda N (2000). Determination of azide in blood and urine by gas chromatography–mass spectrometry. J. Anal. Toxicol..

[13] Ohmori T (2014). High distribution of azide in blood investigated in vivo, and its stability in blood investigated in vitro. Forensic Toxicol..

[14] Kudo K (2018). Reliable determination of cyanide, thiocyanate and azide in human whole blood by GC–MS, and its application in NAGINATA–GC–MS screening. Forensic Toxicol..

[15] Burger E, Bauer HM (1965). Akuter Vergiftungsfall durch versehentliches Trinken von Natriumazidlösung. Arch. Toxicol..

[16] Weiss JS (1996). Reactive airway dysfunction syndrome due to sodium azide inhalation. Int. Arch. Occup. Environ. Health.

[17] Schwarz ES (2014). Multiple poisonings with sodium azide at a local restaurant. J. Emerg. Med..

[18] Gussow L (2010). The case of the contaminated coffee pot. Emerg. Med. News.

[19] Edmonds OP, Bourne MS (1982). Sodium azide poisoning in five laboratory technicians. Br. J. Ind. Med..

[20] Gordon SM (1990). Epidemic hypotension in a dialysis center caused by sodium azide. Kidney Int..

[21] Gaulier JM (2012). Sodium azide intoxications: About two cases. Ann. Toxicol. Anal..

[22] French LK, Hendrickson RG, Horowitz BZ (2012). Sodium azide ingestion associated with QRS prolongation. Abstracts of the 2012 International Congress of the European Association of Poisons Centers and Clinical Toxicologists, 25 May–1 June 2012, London, UK. Clin. Toxicol..

[23] Leonard JB, Quaal-Hines E, Anderson BD (2020). Prime eligible poisons: identification of extremely hazardous substances available on Amazon.com^®^. Clin. Toxicol..

[24] Scientific Working Group for Forensic Toxicology (SWGTOX) (2013). Standard practices for method validation in forensic toxicology. J. Anal. Toxicol..

[25] Peters FT, Drummer OH, Musshoff F (2007). Validation of new methods. Forensic Sci. Int..

[26] Chambers E, Wagrowski-Diehl DM, Lu Z, Mazzeo JR (2007). Systematic and comprehensive strategy for reducing matrix effects in LC/MS/MS analyses. J. Chromatogr. B.

[27] Wachełko O, Szpot P, Zawadzki M (2020). A novel simple and precise method for the determination of azide impurity in sartans using headspace gas chromatography with two dissimilar capillary columns and two flame ionization detector (HS-GC-FID/FID). J. Pharm. Biomed..

[28] Baselt RC (2017). Disposition of Toxic Drugs and Chemicals in Man.

